# Improved endoscope for imaging and cell collection in the fallopian tubes

**DOI:** 10.1117/1.BIOS.3.2.025001

**Published:** 2026-03-17

**Authors:** Dominique Gálvez, Andrew Daniel Rocha, Alana G. Gonzales, Dilara J. Long, Photini Faith Rice, Makayla R. Johnson, Ethan Tovar, Bo Lwin, Helen Varghese, Kongit Amaha, David Fishman, John M. Heusinkveld, Jennifer Kehlet Barton

**Affiliations:** a University of Arizona, Wyant College of Optical Sciences, Tucson, Arizona, United States; b University of Arizona, Department of Biomedical Engineering, Tucson, Arizona, United States; c New York Presbyterian Queens Hospital, Queens, New York, United States; d University of Arizona, Department of Obstetrics and Gynecology, Tucson, United States

**Keywords:** endoscopy, endoscope, ovarian cancer, fluorescence, biopsy, fallopian tubes.

## Abstract

**Significance:**

Early detection of ovarian cancer requires the observation of subtle changes within the fallopian tubes, where serous tubal intraepithelial carcinoma lesions, the putative precursor of high-grade ovarian serous carcinoma, may be present and detectable.

**Aim:**

The cell-acquiring fallopian endoscope was designed to detect and interrogate potentially pathological sites in the fallopian tubes via alterations in fluorescence signal and the collection of epithelial cells.

**Approach:**

We performed a comprehensive redesign of a first-generation endoscope prototype and demonstrated its performance in whole, human *ex vivo* fallopian tubes. Through the iterative improvements to the design, the endoscope features improved imaging, cell collection, and general ease of use. Specifically, improvements included a larger core count fiber imaging bundle, a redesigned close-focus lens, more flexible materials, an altered cell collection method, and a lighter-weight handle.

**Results:**

With benign fallopian tube samples from three patients, we demonstrate cell collection on the order of $10^{4}$ cells per collection and imaging capabilities that result in average image intensity ratios.

**Conclusions:**

This second-generation endoscope is suitable for the study of intact fallopian tubes.

Statement of Discovery A submillimeter cell-acquiring fallopian tube endoscope was designed to use reflectance and fluorescence imaging to detect the presence of precursor lesions of high-grade ovarian serous carcinoma. This could lead to a minimally invasive screening method for early signs of ovarian cancer.

## 1 Introduction

Among gynecological cancers, ovarian cancer has the lowest one-, three-, and five-year survival rates.^1^ This lethality is due to the combination of vague, nonspecific symptoms and the lack of a screening methodology to detect early-stage disease.^2^ With contemporary methods of ovarian cancer detection, most cases are diagnosed with widespread disease and advanced-stage cancer, where the 5-year survival rate can be as low as just 30.8%. In comparison, when a diagnosis is made while the disease is in an early-stagelocalized and confined to the ovary—the survival rate jumps to 91%.^1^ Therefore, the development of a novel detection method of early-stage disease has great potential to reduce mortality from ovarian cancer.

The majority of ovarian cancers are classified as epithelial carcinomas, with most being serous cancers.^3^ In many or most cases, it is believed that high-grade serous carcinoma originates in the fallopian tubes (FTs) as serous tubal intraepithelial carcinoma (STIC) lesions.^4^^–^^8^ These precursor lesions may be present and detectable for several years prior to the development into ovarian cancer.^7^^,^^9^^,^^10^ STIC lesions are clusters of cytologically premalignant cells that are typically present at the distal portion of the fallopian tube.^11^^,^^12^ During malignant transformation, there are changes in the extracellular matrix, metabolism, and vascularization of the tissue,^10^^,^^13^^,^^14^ resulting in expected alterations in optical signals, including reflectance and autofluorescence imaging.^10^^,^^15^ The STIC lesions and subsequent ovarian cancer may be targetable by an exogenous fluorophore due to changes in the quantity and availability of cell surface receptors and extracellular proteins.^16^^,^^17^ Therefore, fluorescence techniques hold strong promise for early-stage cancer detection. In addition, STIC lesions are identifiable through cytological or omics means due to their distinct molecular and cellular signatures.^7^^,^^8^^,^^13^^,^^14^ Therefore, a device that can image the FT and collect cells could potentially enable detection of early-stage ovarian cancer.

Imaging falloposcopes were first developed in the 1990s^18^; however, they were white light illumination only, and imaging resolution was limited by the couple thousand element fiber bundles. A scanning fiber endoscope^19^ has more recently demonstrated imaging of the fallopian tubes; however, it could not consistently cannulate the FTs. Our group performed a pilot study of a falloposcope with both fiber bundle imaging and optical coherence tomography.^20^^,^^21^ A commercially available device, the FemDx FalloView^22^ can provide white light imaging with a chip-on-tip approach, but the large size can limit imaging to the proximal tube only. None of these endoscopes incorporated cell collection. Our previous work resulted in a first-generation prototype cell-acquiring fallopian tube endoscope (CAFE), a minimally invasive, submillimeter diameter endoscope capable of reflectance and fluorescence imaging, as well as cell collection.^23^ The first iteration of the endoscope was tested in small biopsies of normal FTs, where it met the desired imaging and cell collection requirements. However, multiple areas of performance improvement were noted by physician and scientists. Here, we introduce a completely redesigned CAFE. This new endoscope was tested in intact, surgically removed whole human fallopian tubes, demonstrating suitability for eventual *in vivo* use.

## 2 Design Goals

The mechanical, optical, biological, and operational design goals for this second-generation endoscope are listed below in Table 1. These requirements reflect lessons learned during testing of the first-generation endoscope and include increased flexibility, decreased working distance, increased resolution, color imaging, and a reduced weight. Although the first-generation CAFE was adequate for imaging a small biopsy sample, it was noted that increased flexibility would be needed to traverse sometimes tortuous FTs encountered *in vivo*. In addition, we noted that the narrow lumen of the intact FT can collapse around the endoscope, resulting in tissue being very close to the lensed distal tip of the endoscope. The working distance requirement was shortened to ensure the endoscope formed an in-focus image. The resolution of the first-generation prototype, limited by available fiber bundle technology, as well as the monochromatic images produced by laser illumination sources and a monochromatic camera, were deemed suboptimal. Color imaging for navigation was desired. The large handle size and weight complicated operations; thus, an overall weight limit was added to the specifications.

**Table 1:** Mechanical, imaging, cell collection, and safety requirements, as well as clinical considerations required of the proposed endoscope.

Category	Specification	Requirement	Reason
Mechanical	Outer diameter	≤1 mm	Endoscope must fit through FT isthmus with a diameter as narrow as 1 mm
	Restricted diameter Length	≥11 cm	Traverse fallopian tube length of 11 to 12 cm
	Total insertable length	≥70 cm	Traverse entire reproductive tract
	Flexibility	≤20 mm bend radius	Follow the curvature of the fallopian tube
	Introducing method	Ability to enter and track FT lumen	Avoid catching on tortuous, elastic FT walls or plicae
	Weight	≤100 g	Reduced weight of the device increases ease of use for physicians during the imaging procedure.
Imaging	Resolution	≤20 μm at working distance (best focus)	Visualize STIC lesions as small as 100 μm diameter
	Field of view	≥45 deg	Visualize walls of the FT
	Working distance	≤2 mm in air	Allow endoscope to focus on close walls of the fallopian tube
	Depth of field	≥1 mm	Give user as long of a range as possible in which the image will be focused
	Fluorescence sensitivity	Detect autofluorescence in ≤0.1 s imaging time	Visualize autofluorescence without undue motion artifact
	Illumination wavelength	Narrowband blue	Identify alterations in natural autofluorescence, enhance hemoglobin contrast in reflection
	Illumination wavelength	Narrowband red	Excite red near-infrared exogenous fluorescence agents
	Illumination wavelength	White light	Enable navigation
	Illumination angle	≥45 deg	Illuminate entire field of view
Cell collection	Cellution sample amount	≥100 cells per sample	Fulfill sample size requirement for karyometry and most-omics analyses
Safety	Laser power	≤1.4 mW for our geometry	Meet ANSI standard Z136.1
	System electrical leakage current	≤100 μA under normal working conditions	Meet clinical electrical safety requirement IEC 60601-1
	Tissue damage	Biopsy minimally traumatic	Minimize damage to tissue deep to epithelium, avoid perforation
	Sterilizability	Sterilizable by low temperature hydrogen peroxide	Eventual *in vivo* use requires sterilization by a common method
Clinical considerations	Invasiveness	Office-based or outpatient procedure	Reduce patient morbidity, cost

## 3 System Design

**Fig. 1:**
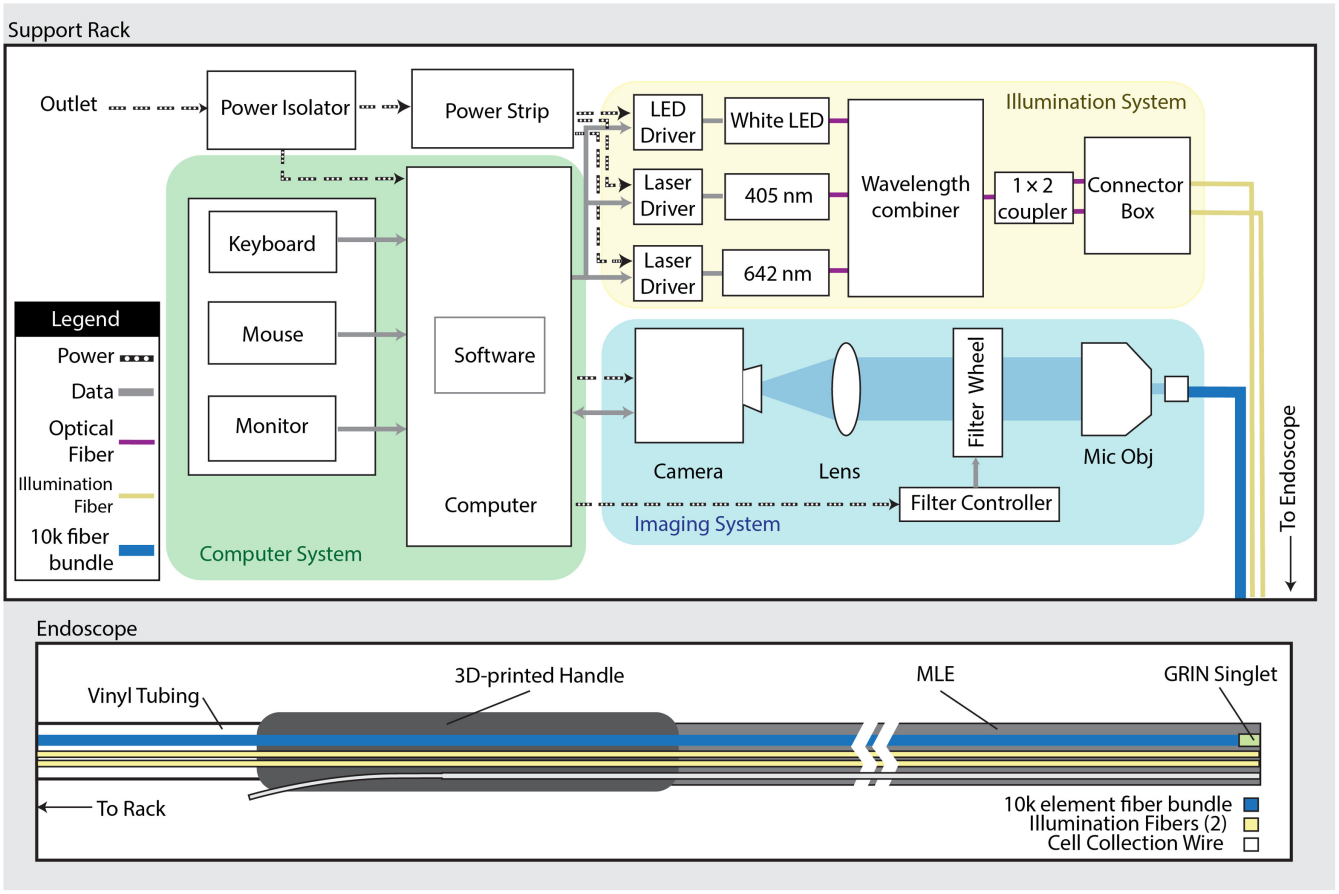
System diagram of the redesigned CAFE. The top portion illustrates the system rack and its contents, including the computer system (green shaded), the illumination system with three light sources (yellow shaded), and the imaging system, which images the fiber bundle onto a camera (blue shaded). The bottom portion displays the endoscope body itself and the components within, including the fiber bundle, illumination fibers, and cell collection wire. [“Mic Obj”: microscope objective; “MLE”: multilumen extrusion; “GRIN”: gradient refractive index; “LED”: light-emitting diode].

The CAFE system was designed to meet the requirements imposed in the section above and to prioritize the feedback given by physicians to improve ease of use when compared with previous fallopian tube endoscopes.^19^^,^^20^ In additionally, changes were made to decrease assembly complexity and improve robustness. The CAFE system consists of two main subsystems, as shown in Fig. 1: the support rack and the endoscope itself.

### 3.1 Support Rack

The support rack (PTRK-14, Middle Atlantic) contains all proximal electrical, optical, and mechanical components. A medical-grade power isolator (IS1800HG, Tripp-Lite, Chicago, Illinois, United States) protects and powers all components.

#### 3.1.1 Illumination subsystem

The illumination subsystem consists of three light sources: 405 nm laser driver and diode (CLD1011LP, LP405-SF10, Thorlabs, Newton, New Jersey, United States), 642 nm laser driver and diode (CLD1010LP, LP642-SF20, Thorlabs), and a white light driver and LED (DC2200, MCWHF2, Thorlabs). The measured values of the nominally 405 and 642 nm lasers are center wavelengths of 404.5 and 642.7 nm, respectively, and full-width half-maximum spectral linewidth of <1  nm. The three fiber-coupled sources are combined in a custom wavelength combiner module (Castor Optics) before splitting into two FC/PC output ports (ADAFC1, Thorlabs) via a custom 1×2 fiber coupler (Castor Optics).

#### 3.1.2 Imaging subsystem

The imaging subsystem begins at the SMA input port (SM1SMA, Thorlabs), where the distal end of the endoscope’s fiber bundle is imaged by a 20X infinity corrected microscope objective (1-U2B225, Olympus, Tokyo, Japan). The collimated light propagates through a filter slider system (ELL9K, Thorlabs), where the filter choices include blank (WG11050-A, Thorlabs), 450 nm long-pass filter (FELH0450, Thorlabs), or 655 nm long-pass filter (ET655lp, Chroma, Taoyuan City, Taiwan). These three filters are used for reflectance imaging at all wavelengths, 405 nm fluorescence imaging, or 642 nm fluorescence imaging, respectively. The fiber bundle image is then focused by an achromatic lens (32-494, Edmund Optics, Barrington, New Jersey, United States) onto a high-sensitivity color camera (Micropublisher 6, Teledyne Vision Solutions, Ontario, Canada). The optical density of the 450 and 655 nm long pass filters at their respective laser excitation wavelengths is 5.24 and 6.02, respectively,^24^^,^^25^ and imaging of diffuse-finish metal (nonfluorescent) targets confirmed negligible laser light leakage to the camera, even at 1 s exposure times.

#### 3.1.3 Computer subsystem

The computer subsystem consists of a Windows OS computer (HP Z2 Mini G4 Workstation, HP) with keyboard, mouse, and monitor located on top of the rack. The computer simultaneously runs two programs: 1) ELLO (Elliptec, Thorlabs) for controlling the filter slider and 2) OCULAR (Teledyne Vision Solutions) to control the camera and display images in real time.

### 3.2 The Endoscope

The endoscopes are designed to be interchangeable and “plug and play” into the support rack. The endoscope consists of three sections, from distal to proximal: 1) the insertable portion, 2) the handle, and 3) the umbilical to rack.

#### 3.2.1 Insertable portion

**Fig. 2:**
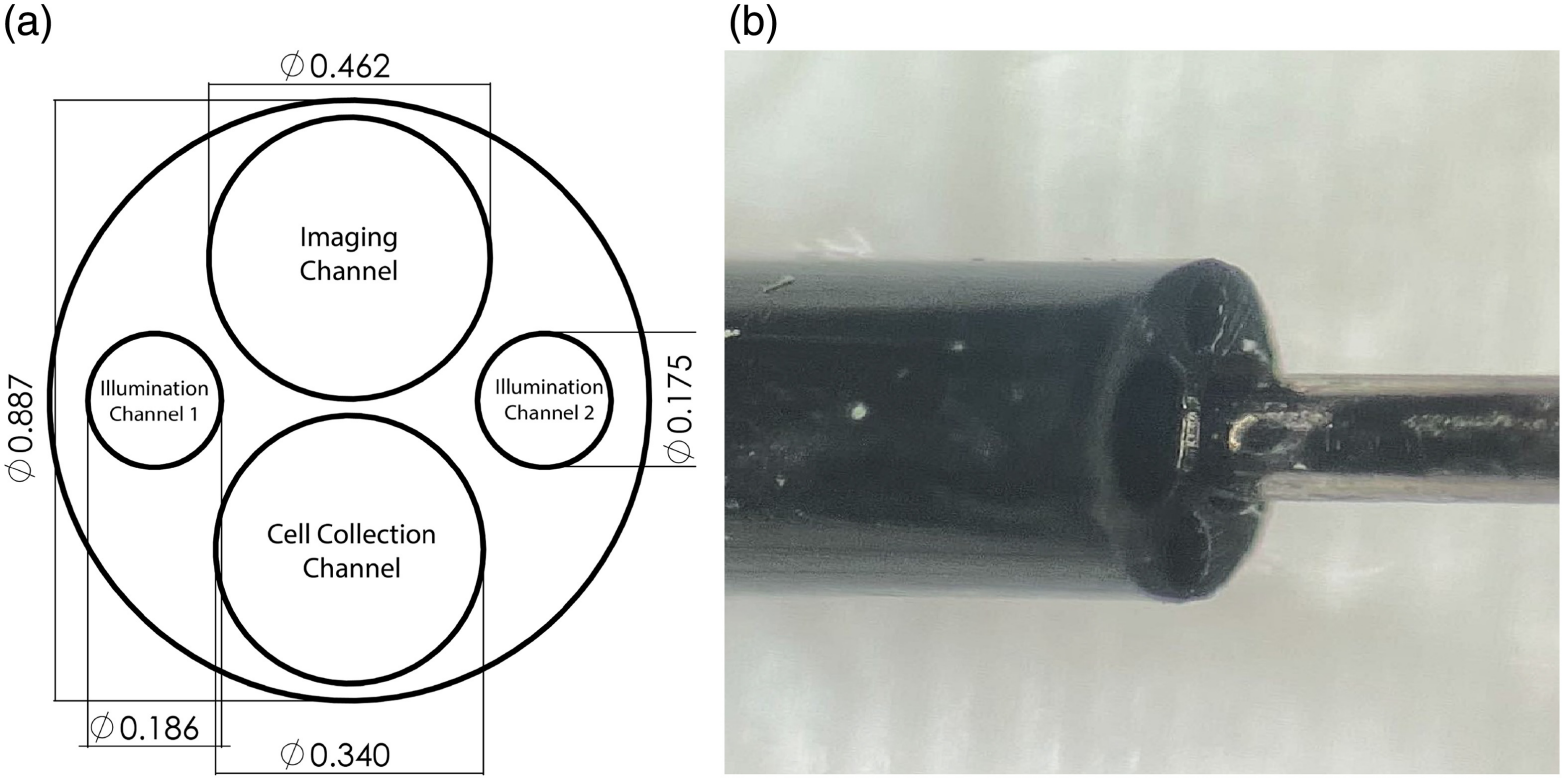
(a) Cross-sectional view of the MLE design, units in mm, with channel functionality labeled. (b) Picture of the as-built MLE face with a stainless-steel wire protruding from the cell collection channel.

The working length of the insertable portion is a custom Pebax multilumen extrusion (MLE) (GenX Medical) with four channels for housing the illumination subsystem, imaging subsystem, and a cell collection wire, as shown in Fig. 2.

##### 3.2.1.1 Distal ferrule

**Fig. 3:**
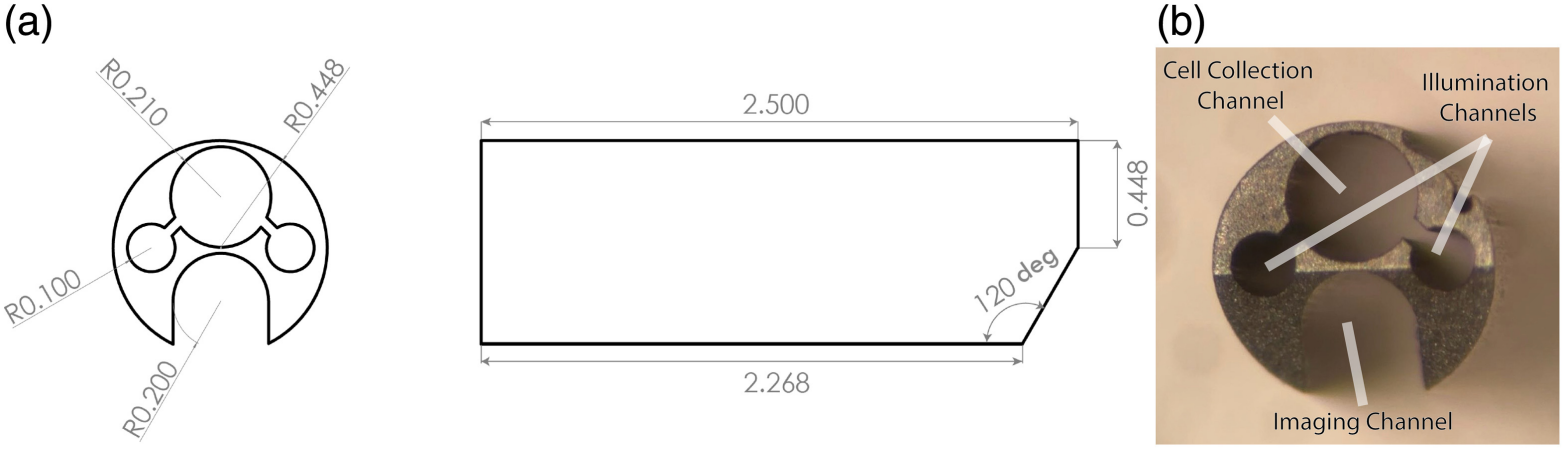
(a) Specifications diagram of the stainless steel distal-tip ferrule (units in mm) and (b) an *en face* microscope image of the as-built ferrule with channel functions labeled.

In previous endoscope designs, fibers and lenses were epoxied directly into the MLE; however, for increased robustness, a custom 2.5 mm-long stainless-steel ferrule (Majer Precision) is utilized. The specification diagram of the ferrule, and *en face* as-built view, are shown in Fig. 3. It serves as both an endcap to the MLE and as a stable surface for securing all the components at the distal tip. To make navigating the FTs easier than in previous endoscope designs, the ferrule features an angled front face. The angled front face, with the guidewire channel leading at the most distal end, enables easier navigation through the tubes than with the blunt MLE surface, which may catch against FT plicae. The illumination fibers and imaging system are adhered into the ferrule with a medical-grade, biocompatible cyanoacrylate adhesive (Loctite 4013, Henkel Adhesives). Then, a layer of black, polyester heat shrink (103- 0513, Nordson) is shrunk around the distal tip of the endoscope, sealing the optical subsystem from the outside and protecting it from stray light.

##### 3.2.1.2 Illumination subsystem

The illumination subsystem consists of two off-the-shelf, high-NA, multimode fibers (Optran Ultra WFGE, Ceramoptec, Bonn, Germany) with a 105  μm core, 125  μm cladding diameter, and 0.37 NA that connect to the system rack through two FC/PC connectors. Note that in Fig. 2, the illumination channels are offset from the imaging channel, so a straight-polished fiber’s illumination spots would not be centered in the field of view of the imaging system. To provide optimized illumination, the fibers are angle-polished to 23.0 deg, which causes the light cone to be emitted at a 10.8 deg tilt from fiber face normal in an aqueous environment. Without this deflection, the full field of illumination covers about 43.4 deg, but due to the 23.0 deg angle polish of the fiber face, the illumination spot spreads to ∼50.0  deg of illumination. The fibers are rotationally aligned to direct the illumination cone toward the center of the imaging channel.

##### 3.2.1.3 Imaging subsystem

**Fig. 4:**
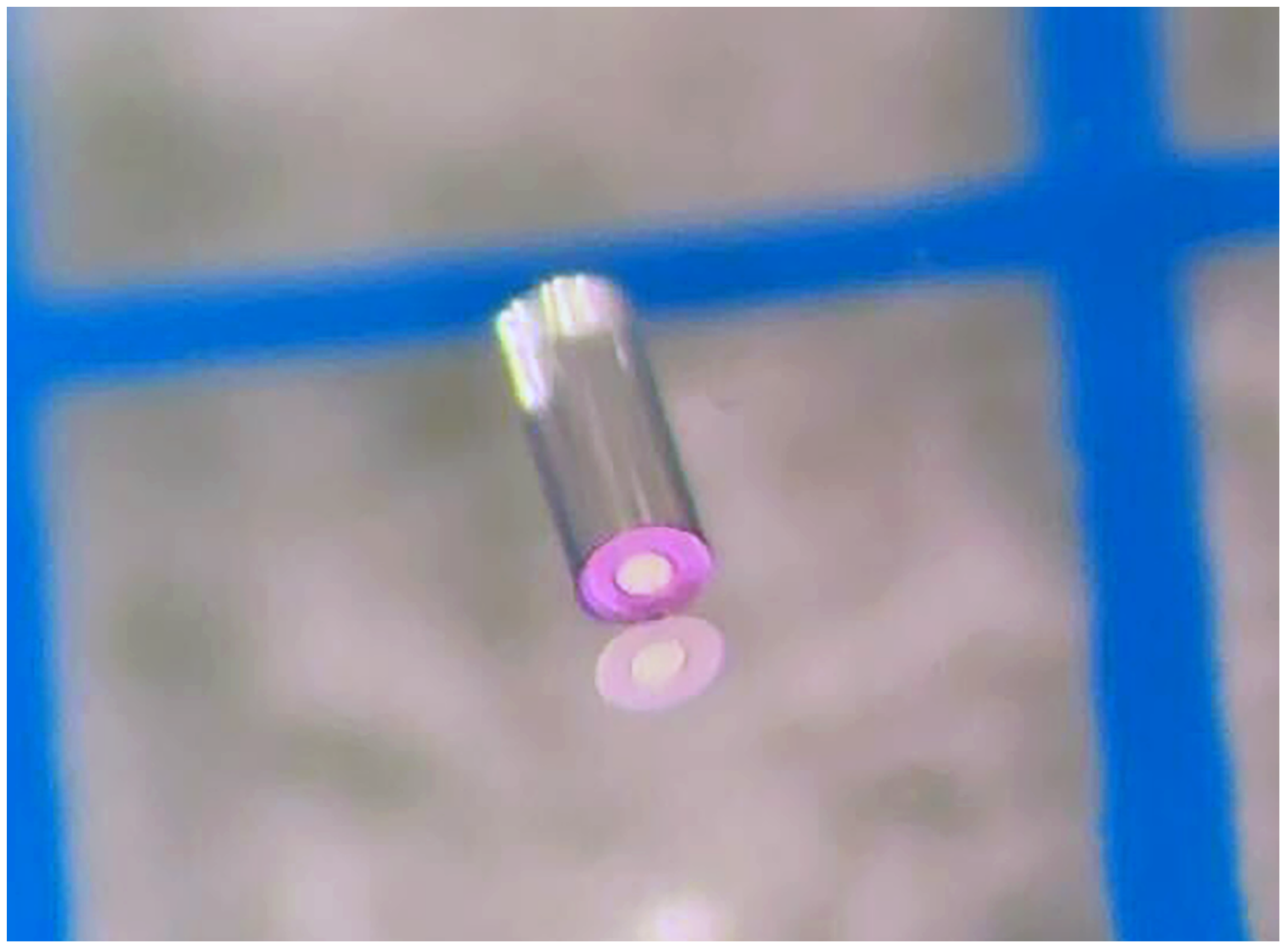
Microscope image of the custom 360  μm diameter lenses, with the black chromium aperture shown (appears pink in image).

The imaging subsystem is composed of an ∼2.5  m-long, 10,000 element fiber bundle (HDIG10K, Sumita, Kanagawa, Japan.) and a custom-pitch 360  μm diameter GRIN rod lens with a 150  μm clear aperture lithographically applied black chromium stop (GT-IFRL-036-0015-50-AS15, GRIN Tech, JenaGermany) on the distal tip. A microscope image of the GRIN rod lens is shown in Fig. 4. The pitch was adjusted in Zemax OpticStudio (Ansys) to achieve a working distance of ∼1  mm in air. This lens was chosen after an exploration of new close-focus lens technologies.^26^ During the design of this endoscope, other designs were attempted, including a custom-pitch, 500  μm diameter GRIN rod lens with a blackened stainless-steel microwasher aperture that was hand-aligned and cemented. This solution frequently suffered from ghost images due to uneven application of glue and was an extremely time-consuming step in the construction process. By lithographically applying the aperture, we were able to remove this laborious step from the building process as well as achieve more consistent optical results.

To build the imaging subassembly, the ferrule is positioned with the imaging channel U-groove vertical. An insulin needle is used to apply a small droplet of refractive index matching gel (9030, Norland, Nebraska, United States) to the U-groove. The lens is placed into the U-groove and aligned with the ferrule’s distal end before the fiber bundle is pushed into the U-groove from the ferrule’s proximal end until it is flush against the rear surface of the lens. Capillary action wicks the index-matching gel into the narrow interface between the fiber bundle and the lens, providing an optimized optical interface and preventing subsequent glue infiltration. Another needle is used to apply a few larger drops of medical-grade cyanoacrylate (Loctite 4013, Henkel Adhesives) onto the subassembly of the fiber bundle, the lens, and the ferrule. The glue wicks into spaces between the optics and the ferrule not occupied by index-matching gel, securing the optical components to the ferrule.

##### 3.2.1.4 Cell collection device

Where the illumination and imaging channels of the MLE have components permanently fixed within them, the cell collection channel is left unobstructed as it is the working channel of the device. A 0.012″ stainless steel wire is threaded through the working channel, from the proximal handle to the distal tip of the endoscope, and positioned flush with the distal tip entrance to block the channel when cells are not actively being collected. To collect cells, the wire is retracted, leaving the most distal 1 to 2 cm portion of the working channel empty. Gentle rubbing of the endoscope tip against the FT walls dislodges epithelial cells that are then collected into the empty channel.

In addition to the cell collection functionality, the working channel can also be used for navigational purposes. The channel is designed to accommodate an up to 0.14″ commercially available guidewire, such as the Terumo Glidewire (GA1418, Terumo Interventional Systems, Somerset, New Jersey, United States). The guidewire can traverse the often-tortuous FTs first, and then the endoscope can be slid forward along the guidewire (“over-the-wire” technique). The guidewire is then retracted 1 to 2 cm as previously described, if cell collection is desired.

#### 3.2.2 Handle

**Fig. 5:**
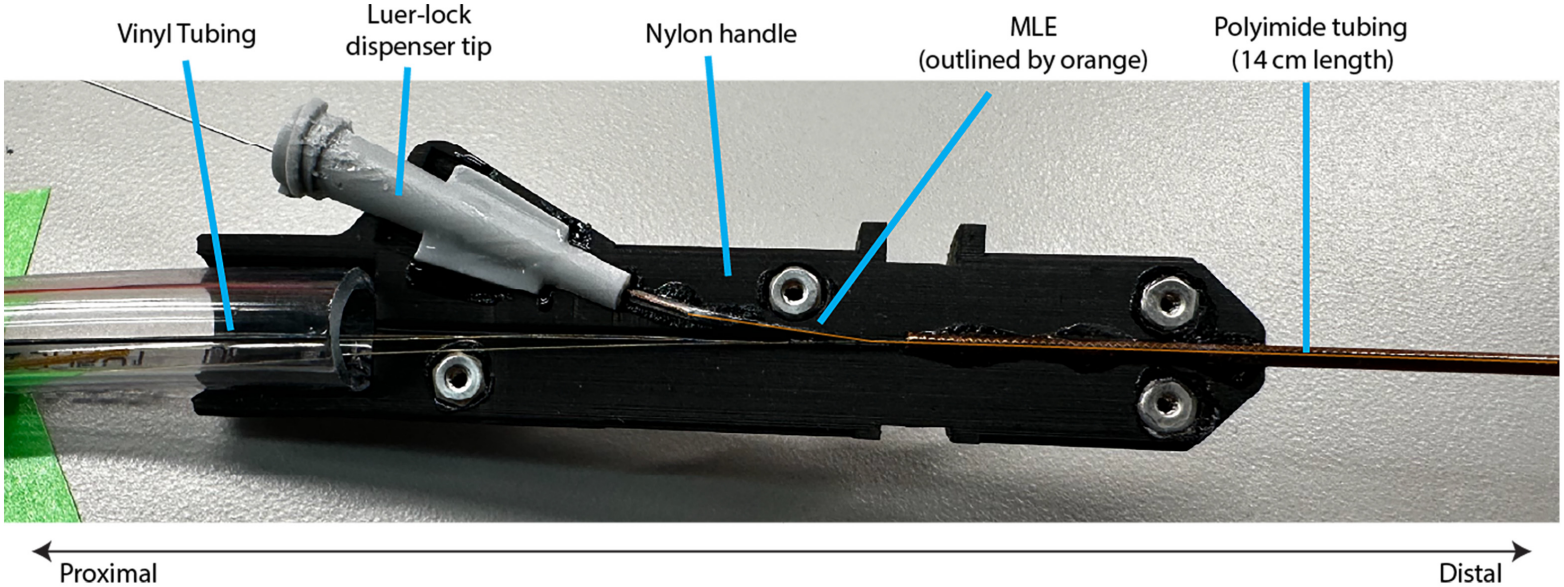
View of one-half of the CAFE handle, with the protective proximal vinyl tubing, modified Luer-lock dispenser tip, nylon handle, MLE, and protective distal polyimide tubing labeled.

A custom, 3D-printed nylon handle allows the operator to safely and securely manipulate the device. Within the handle, the illumination fibers and fiber bundle exit the proximal end of the MLE and enter the larger diameter, more-robust vinyl tubing that protects the fibers traversing to the support rack. On the proximal end of the MLE, a razor blade is used to carefully cut away the illumination and imaging channels, resulting in a 1 to 2 cm length of MLE with the cell-collection channel isolated. A 0.014″ stainless steel wire is threaded through the isolated cell collection channel to serve as an alignment tool so that a 0.0162″ ID polyimide tubing (160- 10, MicroLumen) can be pressed flush against the cell channel opening. The tubing and the isolated MLE channel are then shrunk together with ∼1  cm of polyester heat shrink (103- 0454, Nordon Medical) before the proximal end of the tubing is glued into a custom, 3D-printed modified Luer-lock dispenser tip. These sub-components are displayed and labeled in Fig. 5. This dispenser tip enables wires to be back-loaded into the device or syringes to be attached to flush saline or cleaning solutions through the working channel.

## 4 Experimental *Ex Vivo* Procedure

The ultimate intention for this redesigned CAFE is to perform *in vivo* imaging and cell collection during an outpatient procedure. As a first step toward *in vivo*, we performed a feasibility test of the ability of the endoscope to image whole, freshly removed FTs. In the previous cell acquiring endoscope, imaging was only done on small biopsies. The very small diameter, floppy, tortuous, and plicae-filled FT represent a significantly increased challenge.

Before the imaging procedure, the endoscope was preloaded with 0.012″ stainless steel wire, with the wire’s distal tip positioned flush to the distal tip of the endoscope. Upon receiving the excised tissue, the FT lumen was located and flushed with saline. The endoscope was inserted into the distal, fimbriated end of the FTs because the proximal end was electrocoagulated during the surgical procedure.

**Fig. 6:**
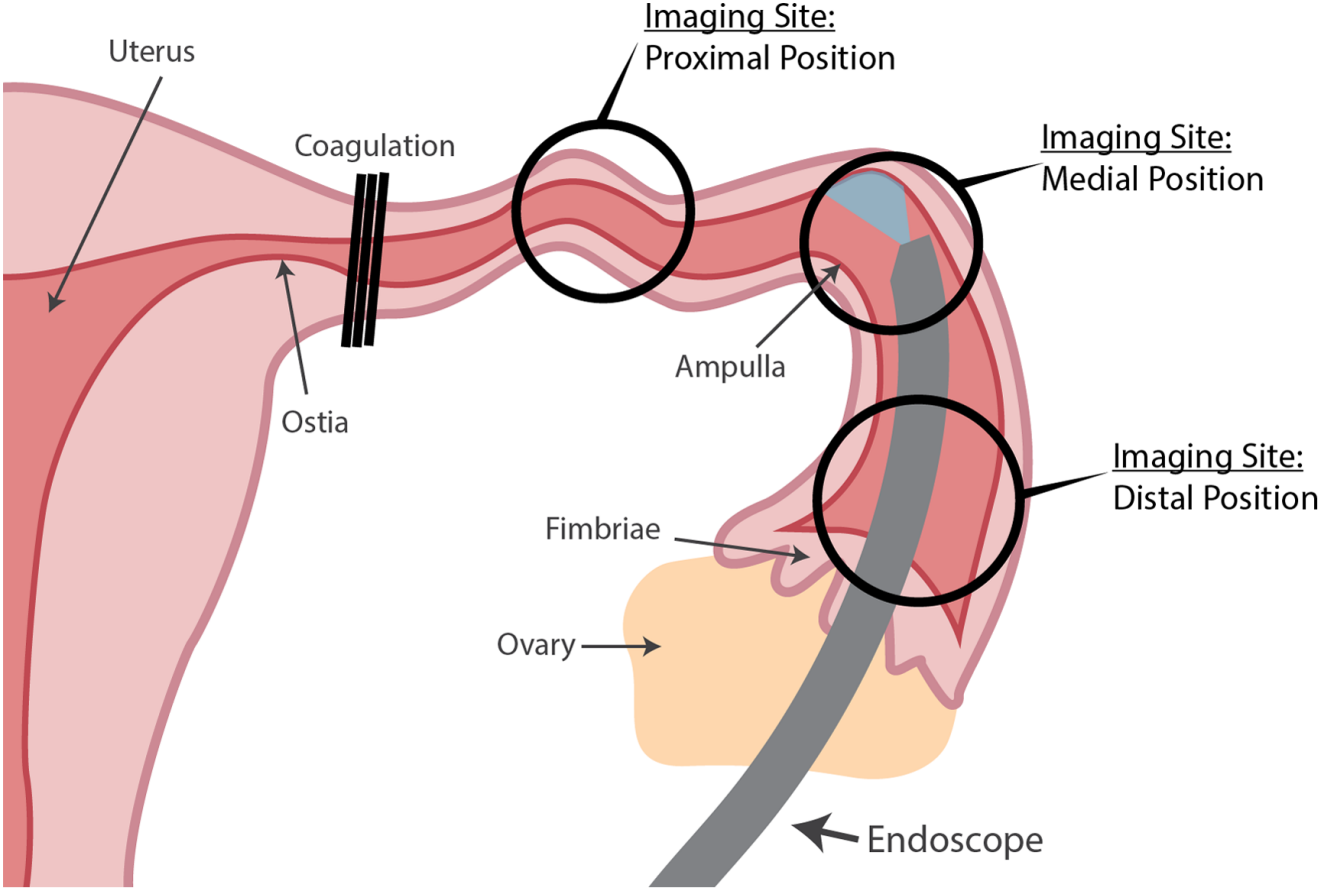
Labeled diagram of the FTs and adjacent structures, with the three imaging sites (proximal FT, medial FT, and distal FT) circled on the diagram. The endoscope was inserted from the fimbriated end for this study, due to the coagulation during surgery of the very proximal FT.

Three sets of images were collected from inside the FTs, at the proximal (approximately 1 cm distal of the uterotubal ostia), medial (approximately located at the ampulla of the FT), and distal (∼0.5  cm proximal from the fimbriae) positions. A diagram of the fallopian tube with the three imaging positions labeled is given in Fig. 6. At each position, four images were collected: white-light reflectance (white light LED with blank filter), 642 nm reflectance (642 nm laser diode with blank filter), 405 nm reflectance (405 nm laser diode with blank filter), and 405 nm fluorescence (405 nm laser diode with 450 nm long pass filter). The 642 nm fluorescence image was not collected in this study because no exogenous dyes were utilized, and autofluorescence is minimal in the red wavelengths. To promote consistency throughout the experiment, the white light LED is set to 100% power, corresponding to ∼0.2  mW of illumination power out of the distal tip, and for the laser reflectance and fluorescence images, the lasers were set to a current producing 1.4 mW of illumination power out of the endoscope. The camera was set to 100 ms exposure. For each image, the operator recorded the patient case number, the left or right FT, the position in the FT, the image type (reflectance or fluorescence), the wavelength, and the exposure time.

## 5 Data Processing and Analysis

Absolute average intensities of the images obtained varied greatly depending upon tissue geometry and endoscope-to-tissue distance. Therefore, ratios of average image intensities were calculated at each location in the FT, similar to previous work.^27^^,^^28^ For each location along the FT, the ratios of the average signal were calculated between 1) 405 nm reflectance and 405 nm fluorescence, 2) white-light reflectance red and blue channels, and 3) white-light reflectance red and green channels.

To do this, the images were masked to include only the fiber bundle image circle. The images from the camera are taken as three-channel, 16-bit RGB images, and are processed differently, depending on if the image is of white light reflectance, or if the image is of 405 nm reflectance or fluorescence.

For 405 nm reflectance and fluorescence images, the 3-channel 16-bit RGB images were averaged into a single-channel 16-bit grayscale image according to the simple averaging given in Eq. (1).

$$\text{Gray Pixel Value}=\frac{\text{Red Pix.}\;\text{Value}+\text{Green Pix.}\;\text{Value}+\text{Blue Pix.}\;\text{Value}}{3}.$$ (1)

**Fig. 7:**
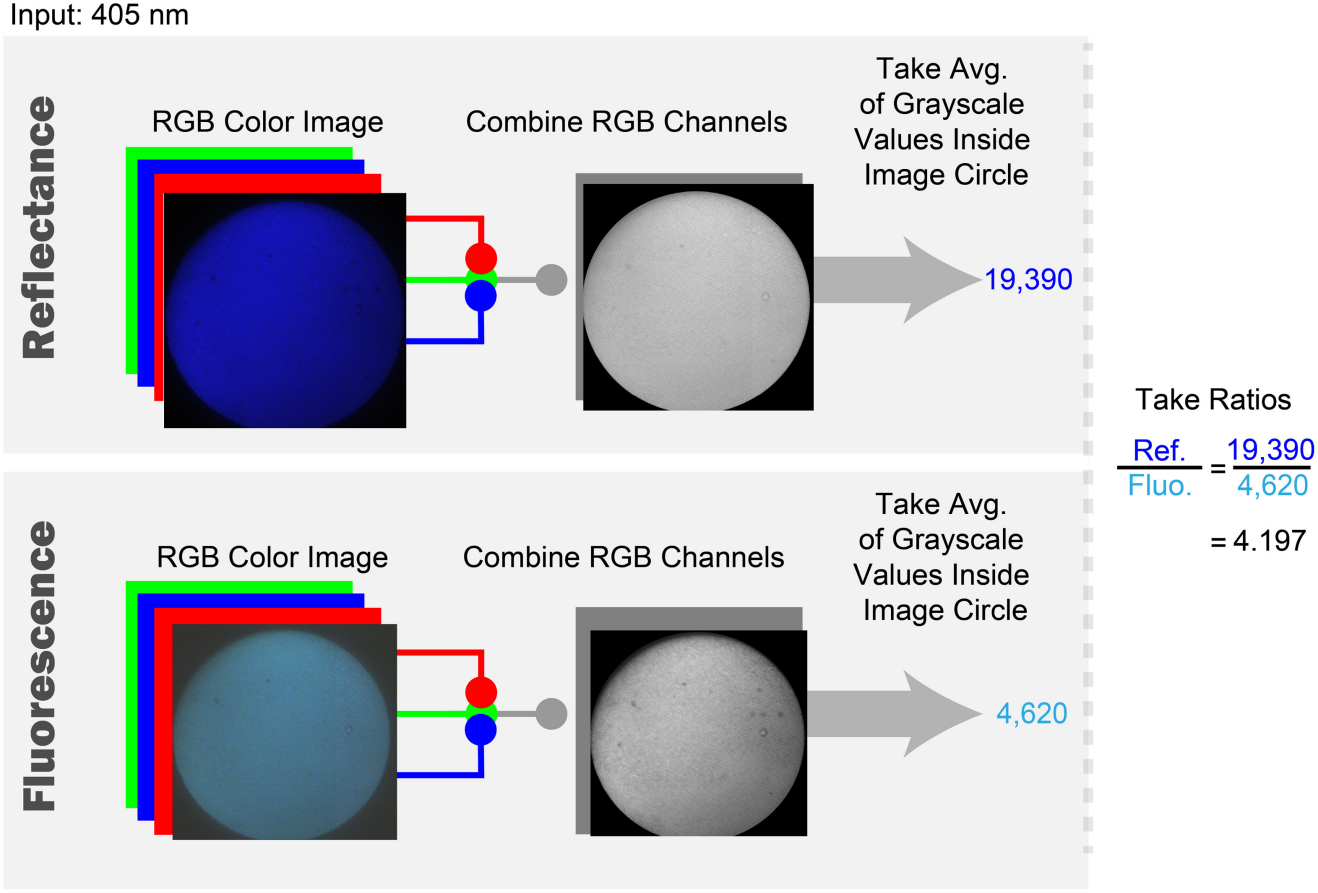
Left-to-right flowchart of the image processing steps for extraction of the average signal values of the reflectance and fluorescence images, and determining their ratio. Numerical values given are examples.

Then, the “average grayscale value” across the entire grayscaled image is calculated, resulting in a single number representing the image signal. Figure 7 depicts the image post-processing steps taken to find the average grayscale values, as well as the reflectance to fluorescence signal ratios.

**Fig. 8:**
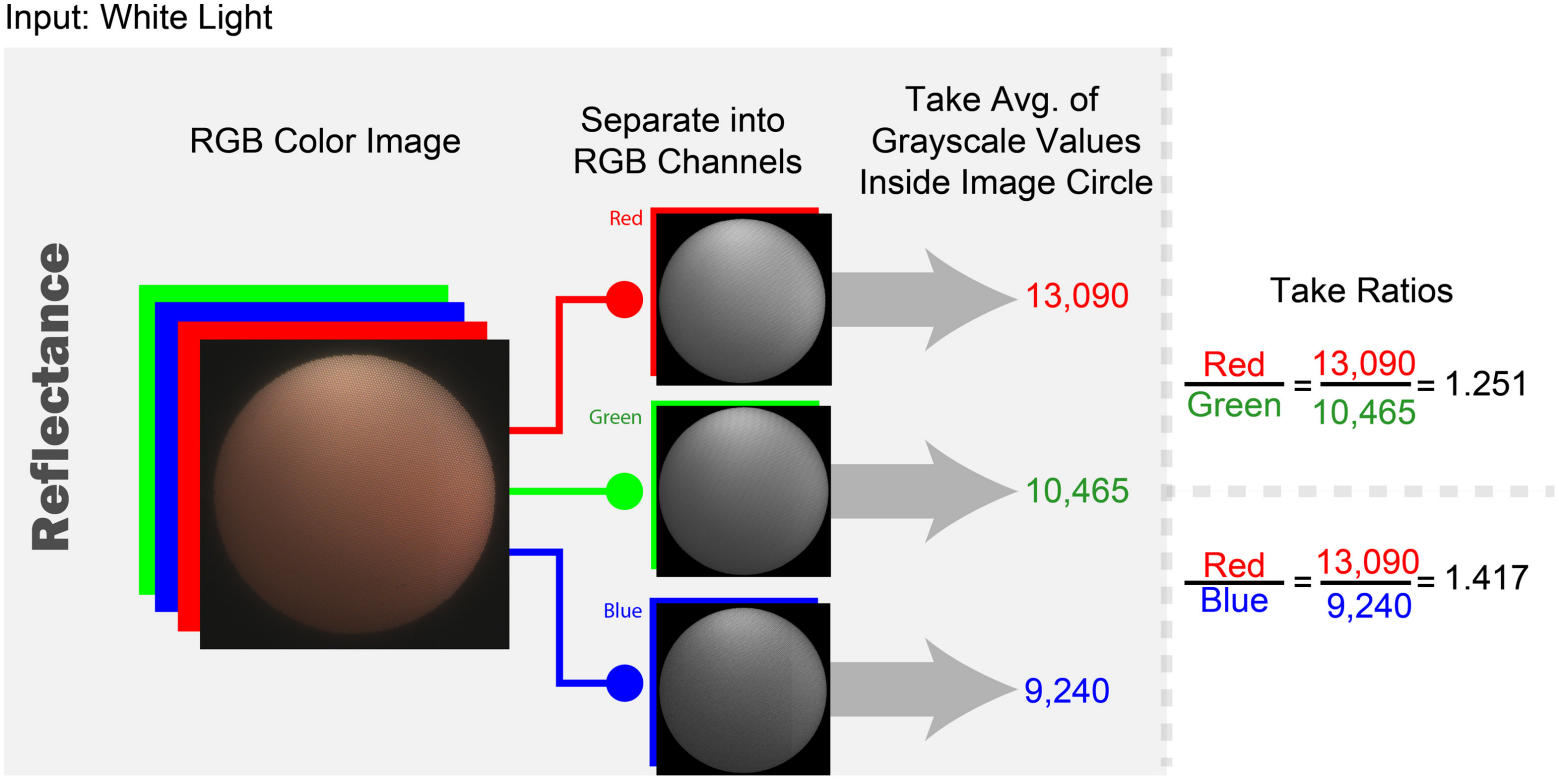
Left-to-right flowchart of the image processing steps for the extraction of the average signal values for the red, green, and blue channels of the white-light reflectance images and determining the red-to-green and red-to-blue ratios. Numerical values given are examples.

For the white light images, the R, G, and B channels of the 16-bit RGB image were separated, and the average grayscale value was found for each separate channel. These channel averages were used to create the red-to-green (R/G) and red-to-blue (R/B) ratios. This process is depicted in Fig. 8.

In addition to the four images collected at each location inside the FT, a white light reflectance pullback video was taken of each FT, as well as white light reflectance, 405 nm reflectance, and 405 nm fluorescence external images of the fimbriae. These videos/images were collected for qualitative visualization of the endoscope’s performance.

## 6 Results and Discussion

Eleven endoscopes were built and tested against the requirements in Table 1 utilizing laboratory setups. Three were used for initial *ex vivo* testing in a hospital setting with tissue from three patients.

### 6.1 Mechanical

All 11 endoscopes met all mechanical requirements, as shown in Table 2. The maximum outer diameter was 0.98 mm, meeting the 1 mm requirement, and length requirements were easily met. The flexibility was acceptable. The 0.012″ guidewire advanced easily through the FT lumen, and the endoscope could be tracked over-the-wire. Feedback from operators, including surgeons, was positive. They found the endoscope to be more robust, flexible, and convenient to handle than previous iterations. The smaller, lighter handle design was appreciated.

**Table 2:** Mechanical requirements and the as-built maxima values of the 11 devices.

Specification	Requirement	As-built
Outer diameter	≤1 mm	0.98 mm
Restricted diameter length	≥11 cm	≥60 cm
Total insertable length	≥70 cm	≥72 cm
Flexibility	≤20 mm bend radius	≤20 mm bend radius
Introducing method	Ability to track FT lumen	Endoscope advanced over-the-wire; guidewire tracks lumen
Weight	≤100 g	15 g

### 6.2 Imaging (Benchtop)

The imaging subsystem fulfilled all requirements outlined in Table 1, except the depth of field. As-built performance is listed in Table 3.

**Table 3:** Imaging requirements versus the as-built values of the device.

Specification	Requirement	As-built
Resolution	≤20 μm at working distance of 1 mm in air	10% contrast at Group 6 Element 1, or 7.81 μm
Field of view (FOV)	≥45 deg	60 deg
Working distance (WD)	≤2 mm in air	∼1 mm
Depth of field (DOF)	≥1 mm	0.65 mm (FAIL)
Fluorescence sensitivity	Detect autofluorescence in ≤0.1 s imaging time	Signal detected
Illumination wavelength	Narrowband blue	405 nm fiber diode laser
Illumination wavelength	Narrowband red	642 nm fiber diode laser
Illumination wavelength	White light	White LED
Illumination angle	≥45 deg	50 deg

#### 6.2.1 Resolution, WD, DOF, FOV, distortion

**Fig. 9:**
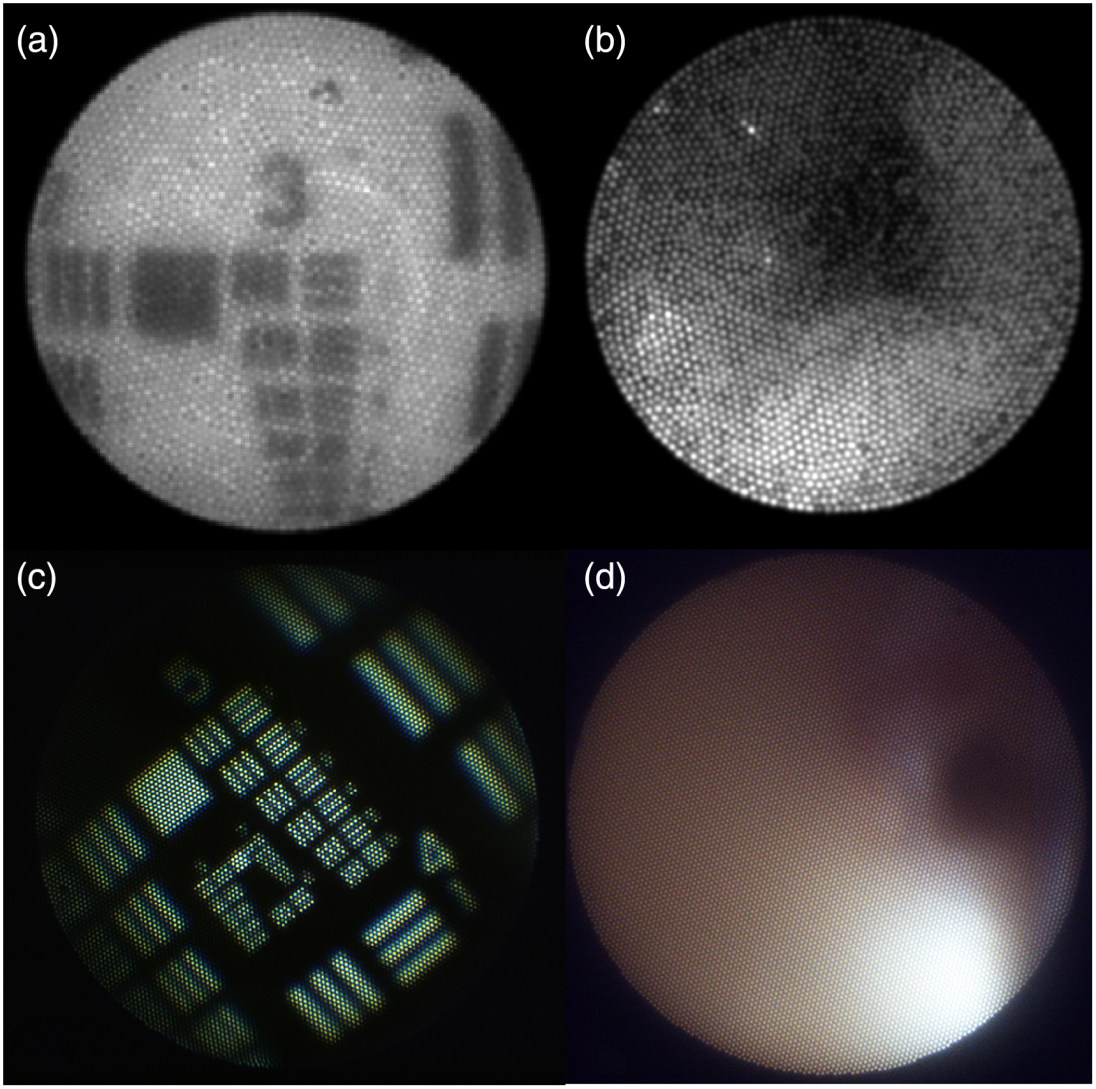
Previous endoscope design. (a) Image of a uniformly illuminated USAF target showing visualization of Group 3 Element 3, viewed at a best-focus distance of 5 mm in air. (b) blue light reflectance image captured during navigation in an *ex vivo* porcine FT. (c and d) New endoscope design. (c) color image of a uniformly back-lit USAF Target showing visualization of Group 6 Element 1 at a best-focus distance of 1 mm in air. (d) Color white-light reflectance image captured during a pullback video through a whole human *ex vivo* FT.

Figure 9 compares the imaging capabilities of the new endoscope versus the previous iteration. The previous system acceptably resolved down to Group 2 Element 4 (corresponding to 88.4  μm) at a best-focus object distance of ∼5  mm. The new system can image down to Group 6 Element 1 (corresponding to 7.81  μm) at a best-focus object distance of ∼1  mm. Note that the object in Fig. 9(a) is a diffuse forward-illuminated positive paper USAF target (PN:38-710, Edmund Optics), while the object Fig. 9(c) is a diffuse back-illuminated negative chromium USAF Target (PN: DA004, MaxLevy/II-VI). This change of resolution targets was necessary because the paper target was limited to Group 3, while the new endoscope can resolve down into Group 6. The higher resolution of the new endoscope is expected, given the larger number of fiber bundle elements and the closer working distance.

The previous design utilized an off-the-shelf GRIN lens with a relatively long working distance (WD) of 5 mm, and a depth of field (DOF) of ∼4  mm – infinity. Unfortunately, in the narrow tortuous lumen of the FT, the walls were much closer than 4 mm, limiting visualization. The new design greatly shortened the WD to ∼1  mm, which inherently shortens the DOF. A contrast of 10% at Group 5 Element 4 is used as the cutoff for determining DOF. With this criterion, the DOF is about 0.65 mm. In the future, efforts can be made to increase the DOF of the system through more complex optical designs, possibly by returning to the exploration of 3D-printed two-photon polymerization lenses as technology rapidly develops.^21^

The full field of view (FOV) increased from ∼45  deg in the previous endoscope design to 60 deg in the new system. This increase in FOV enables a reasonable area of the FT to be visualized at the short WD.

GRIN lenses characteristically have barrel distortion.^29^ Modest distortion is apparent in Figs. 9(a) and 9(c), reaching ∼10% at the maximum FOVs of the two systems. Although barrel distortion causes the apparent size of features to change as a function of field position, it is acceptable in this application because the purpose of the endoscope is visualization, not quantification. It may even be beneficial because objects immediately in front of the endoscope have higher magnification, whereas peripheral awareness of tissue structure is maintained at lower magnification at the edges of the FOV.

#### 6.2.2 Illumination

The angular FOV of the imaging system (∼60  deg) is larger than the angular FOV of the light cone existing our 0.37 NA, angle polished, illumination fibers (∼50  deg.) Therefore, this design utilized two fibers to enable a more uniformly illuminated field. Still, nonuniformity was expected when imaging a flat surface. The ratio of the brightest region to darkest region of an illuminated flat object was taken and found to be ∼10:1. This ratio is comparable to that seen in the previous endoscope (7:1). In practice, and as seen in Fig. 9(d), illumination nonuniformity is not obvious, possibly due to multiple light scattering in the confined FT lumen, and the fact that in tubular organs the tissue at the FOV periphery tends to be closer to the endoscope. In the future, we will explore higher NA fibers, or distal illumination optics to create a larger illumination cone angle. As the illumination pattern is fixed and known, illumination could also be digitally flattened.

### 6.3 Cell Collection

Studies done on three *ex vivo* whole FT samples showed consistent epithelial cell collection between $2.0\times 10^{4}$ and $2.0\times 10^{5}$ cells per collection attempt. This amount is comparable with the previous iteration of the endoscope, which collected on the order of $10^{5}$ cells per collection by extending a curved nitinol wire from the cell collection channel and carefully scraping against the epithelial walls. This current endoscope collects cells directly into the smooth endoscope tip lumen, eliminating the nitinol wire and any concomitant puncture risk.

### 6.4 Safety

The device passes all safety requirements. Electrical safety testing performed by Banner University Medical Center confirmed conformance to electrical leakage requirements, and high-quality images were obtained with allowable laser powers. Sterilization with low-temperature hydrogen peroxide caused no detectable loss in performance. Subsequent histology found no visible damage to the FT from the imaging or cell collection procedure.

### 6.5 *Ex-Vivo* Demonstration

**Fig. 10:**
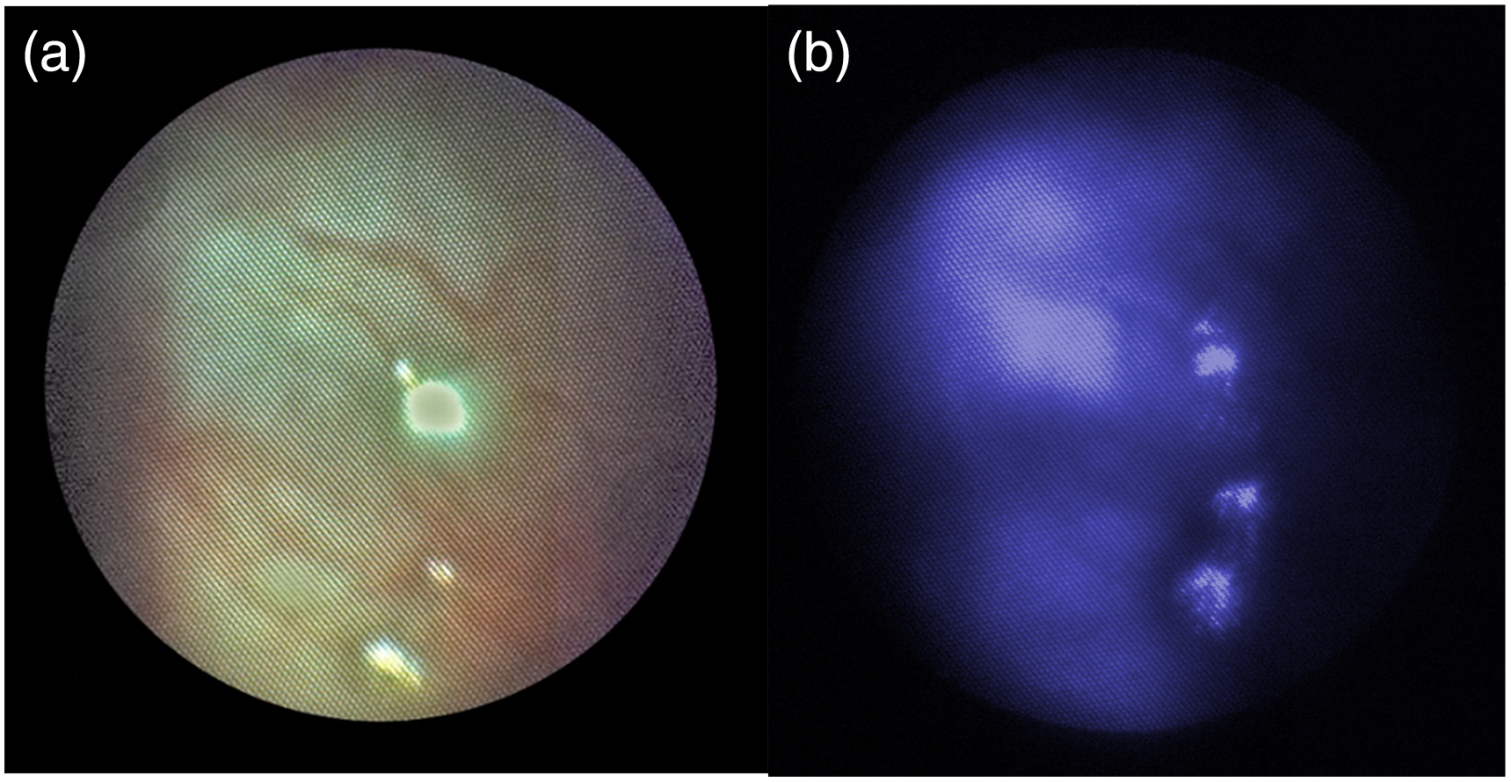
(a) White-light reflectance image, an external view of the fimbriated end of the FT. (b) 405 nm Reflectance image, an external view of the fimbriated end of the FT.

In an initial feasibility demonstration, we imaged the excised FTs of three patients undergoing salpingo-hysterectomy. In each of these samples, the reproductive tracts (uterus and fallopian tubes) were intact, and the tissue was deemed benign by pathology. Images were obtained at all locations per protocol on both the left and right sides, in ∼15  min per patient. An example still from a white light video is shown in Fig. 9(d). Figures 10(a) and 10(b) show example images obtained of the fimbriated end of the FT using white light (a) and blue (405 nm) light reflectance (b). Small blood vessels are clearly seen in the white light images. The blue reflectance image has less contrast, but blood vessels are also apparent as dark linear structures, due to hemoglobin absorption of the blue light. Tissue unevenness resulted in small saturated spots from strong specular reflection, even in an aqueous environment.

**Fig. 11:**
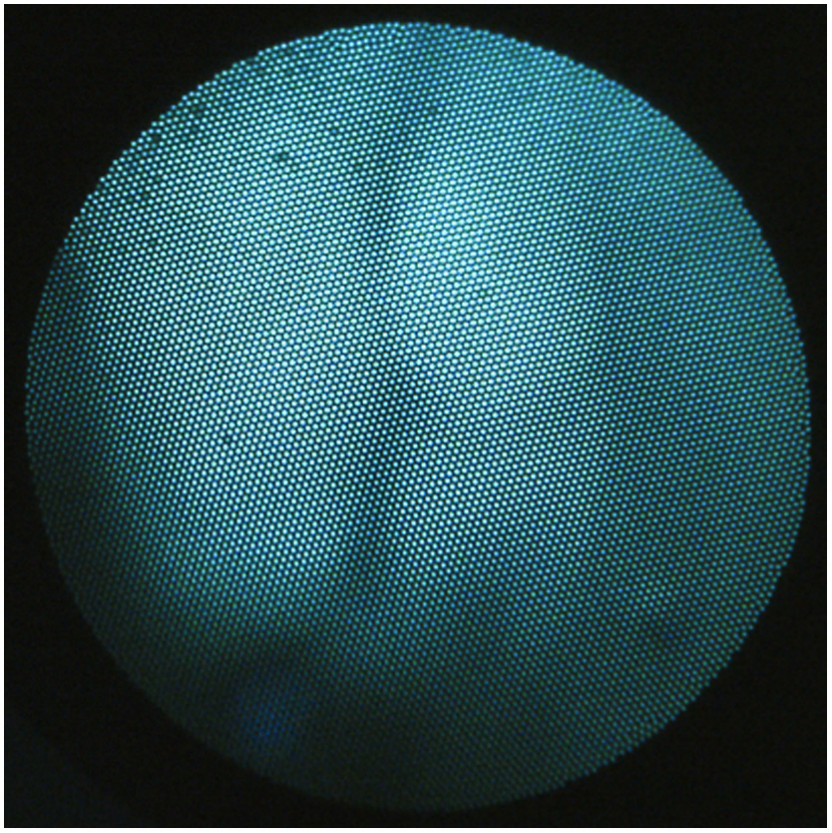
Example 405 nm autofluorescence image of the medial lumen of the FT. Robust signal is captured even at short (100 ms) exposure times. Note that the dark spots in the upper left of the image are due to fiber bundle pixel imperfections.

Figure 11 shows a 405 nm fluorescence image from the medial FT, demonstrating that a robust autofluorescence signal was received even at relatively short (100 ms) exposure times.

**Fig. 12:**
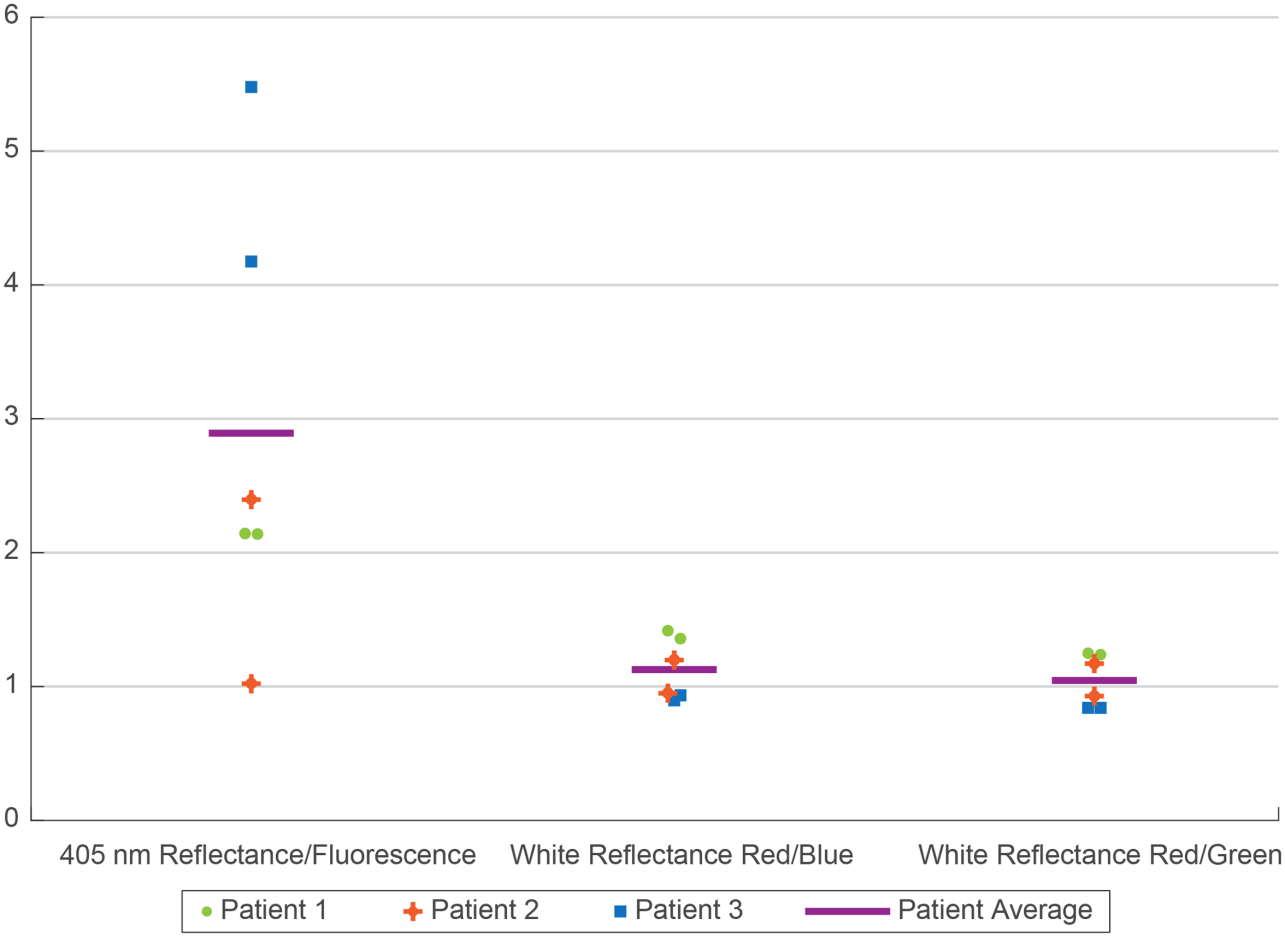
Scatter plot of the average image intensity ratios of two FT each of three patients, (six data points). Three ratios are shown: 405 nm reflectance to 405 nm fluorescence, white-light reflectance red channel to blue channel, and red channel to green channel.

Figure 12 shows average image intensity ratio values for three patients (six FT), all with histologically confirmed benign tissue. Because values obtained at the three locations (distal, medial, proximal) were similar, only the three-location average is shown per FT, and the two FT’s data are shown per patient. Ratio values from the right and left fallopian tubes of the same patient were generally similar, suggesting that the variation in reflectance and fluorescence signals between different patients represents actual inter-patient variability of benign tissue. The 405 nm reflectance to fluorescence image ratios show greater variability between the right and left FTs than the white light red to blue and red to green channel ratios. This variation may be due to slight movement of the endoscope occurring (especially in patients 2 and 3) while switching from reflectance to fluorescence imaging. Differences in endoscope-tissue distance will alter the amount of signal received. In comparison, the white-light reflectance image’s three color channels are taken simultaneously as a single snapshot. Patient 2’s white-light reflectance ratios differ the most between the left and right FT measurements. Although all tissue was confirmed benign, differences in anatomy and tissue composition can occur between the two fallopian tubes, possibly accounting for this dissimilarity.

The results shown in Fig. 12 align with the expectation for benign FT samples. Remitted tissue fluorescence when excited at 405 nm is primarily due to collagen and the metabolic co-factor NADH,^30^^,^^31^ and is orders of magnitude weaker than reflectance. Therefore, 405 nm reflectance/fluorescence ratios are greater than 1. In the visible spectrum, the major tissue absorber is hemoglobin; therefore, ratios of red to blue and red to green light may reflect tissue vascularity and percent blood content. As malignant transformations occur, autofluorescence at 405 nm excitation is expected to decrease.^10^^,^^15^ Further, due to the absorption spectra of hemoglobin, an increase in angiogenesis would lead to an increase in the ratios of R/B and R/G white-light reflectance.^32^^,^^33^

## 7 Conclusion

An improved fallopian tube endoscope was designed and tested. All requirements for mechanical, optical, cell collection, and safety were met, apart from depth of field. In a preliminary demonstration of its utility, we were able to show that the endoscope can enter and image inside the FTs, collect reflectance and fluorescence data, and collect epithelial cells. Continued studies will aim to characterize the variation in fluorescence and reflectance signals from benign FT, as well as examine FT that harbor STIC, cancer, and other pathologies such as salpingitis, to determine if imaging can be used to distinguish benign from pathologic tissue.

## Data Availability

The raw data used to support the conclusions of this paper are included in the Supplemental Materials.

## References

[1] SEER*Explorer: An interactive website for SEER cancer statistics, Surveillance Research Program National Cancer Institute, https://seer.cancer.gov/statistics-network/explorer/application.html?site=1&data_type=4&graph_type=2&comapreBy=relative_survival_interval&chk_relative_survival_interval_1=1&sex=1&race=1&age_range=1&hdn_stage=101&advopt_precision=1&advopt_show_ci=on#resultsRegion0

[2] Olivier R. I. et al., “CA125 and transvaginal ultrasound monitoring in high-risk women cannot prevent the diagnosis of advanced ovarian cancer,” Gynecol. Oncol. 100(1), 20–26 (2006). 10.1016/j.ygyno.2005.08.038 16188302

[3] Torre L. A. et al., “Ovarian cancer statistics,” CA Cancer J. Clin. 68(4), 284–296 (2018). 10.3322/caac.21456 29809280 PMC6621554

[4] Chan A. et al., “New insights into the pathogenesis of ovarian carcinoma: time to rethink ovarian cancer screening,” Obstet. Gynecol. 120(4), 935–940 (2012). 10.1097/AOG.0b013e318269b8b1 22996112

[r5] Shih I. M. Wang Y. Wang T. L., “The origin of ovarian cancer species and precancerous landscape,” Am. J. Pathol. 191(1), 26–39 (2021). 10.1016/j.ajpath.2020.09.006 33011111 PMC7786078

[r6] Vang R. Ie M. S. Kurman R. J., “Fallopian tube precursors of ovarian low- and high-grade serous neoplasms,” Histopathology 62(1), 44–58 (2013). HISTDD 1365-2559 10.1111/his.12046 23240669

[r7] Labidi-Galy S. I. et al., “High grade serous ovarian carcinomas originate in the fallopian tube,” Nat. Commun. 8(1), 1093 (2017). 10.1038/s41467-017-00962-1 29061967 PMC5653668

[8] Perets R. Drapkin R., “It’s totally tubular….riding the new wave of ovarian cancer research,” Cancer Res. 76(1), 10–17 (2016). CNREA8 0008-5472 10.1158/0008-5472.CAN-15-1382 26669862 PMC4703449

[9] Brown P. O. Palmer C., “The preclinical natural history of serous ovarian cancer: defining the target for early detection,” PLoS Med. 6(7), e1000114 (2009).1549-1676 10.1371/journal.pmed.1000114 19636370 PMC2711307

[10] McAlpine J. N. et al., “Autofluorescence imaging can identify preinvasive or clinically occult lesions in fallopian tube epithelium: a promising step towards screening and early detection,” Gynecol. Oncol. 120(3), 385–392 (2011). 10.1016/j.ygyno.2010.12.333 21237503

[11] Lee Y. et al., “A candidate precursor to serous carcinoma that originates in the distal fallopian tube,” J. Pathol. 211(1), 26–35 (2007). 10.1002/path.2091 17117391

[12] Seidman J. D. et al., “The fallopian tube-peritoneal junction: a potential site of carcinogenesis,” Int. J. Gynecol. Pathol. 30(1), 4–11 (2011). 10.1097/PGP.0b013e3181f29d2a 21131840

[13] Kim J. et al., “Cell origins of high-grade serous ovarian cancer,” Cancers 10(11), 433 (2018). 10.3390/cancers10110433 30424539 PMC6267333

[14] Meserve E. E. K. Brouwer J. Crum C. P., “Serous tubal intraepithelial neoplasia: the concept and its application,” Mod. Pathol. 30(5), 710–721 (2017). 10.1038/modpathol.2016.238 28106106

[15] Tate T. H. et al., “Multispectral fluorescence imaging of human ovarian and fallopian tube tissue for early-stage cancer detection,” J. Biomed. Opt. 21(5), 056005 (2016). 10.1117/1.JBO.21.5.056005 27220626 PMC5996865

[16] Kashima H. et al., “Laminin C1 expression by uterine carcinoma cells is associated with tumor progression,” Gynecol. Oncol. 139(2), 338–344 (2015). 10.1016/j.ygyno.2015.08.025 26343160 PMC4862403

[17] Sawyer T. W. et al., “Quantification of multiphoton and fluorescence images of reproductive tissues from a mouse ovarian cancer model shows promise for early disease detection,” J. Biomed. Opt. 24(9), 096010 (2019). 10.1117/1.JBO.24.9.096010 31571434 PMC6768507

[18] Kerin J. et al., “Warren Grundfest,“Falloposcopy: a microendoscopic technique for visual exploration of the human fallopian tube from the uterotubal ostium to the fimbria using a transvaginal approach,” Fertil. Steril. 54(3), 400 (1990). FESTAS 0015-0282 10.1016/S0015-0282(16)53750-9 2397786

[19] Seibel E. J. et al., “In vivo laser-based imaging of the human fallopian tube for future cancer detection,” Proc. SPIE 9304, 93040Q (2015).

[20] Rocha A. D. et al., “Iterative prototyping based on lessons learned from the falloposcope in vivo pilot study experience,” J. Biomed. Opt. 28(12), 121206 (2023). 10.1117/1.JBO.28.12.121206 37577082 PMC10423010

[21] Rocha A. D. et al., “First clinical feasibility and safety study of a novel multimodality fallopian tube imaging endoscope,” Lasers Surg. Med. 57(2), 163–170 (2025). 10.1002/lsm.23877 39789754

[22] U. S. Food and Drug Administration (FDA), “Premarket approval for femDx falloView,” 510(k): K221965, 2023, https://www.accessdata.fda.gov/scripts/cdrh/cfdocs/cfpmn/pmn.cfm?ID=K221965.

[23] Cordova R. et al., “Sub-millimeter endoscope demonstrates feasibility of in vivo reflectance imaging, fluorescence imaging, and cell collection in the fallopian tubes,” J. Biomed. Opt. 26(7), 076001 (2021). 10.1117/1.JBO.26.7.076001 34216135 PMC8253554

[24] Thorlabs, “FELH0450 optical density,” Hard-Coated Edgepass Filters, www.thorlabs.com.

[25] Chroma, “ET655lp,” https://www.chroma.com/products/parts/et655lp.

[26] Galvez D. et al., “Characterizing close-focus lenses for microendoscopy,” J. Opt. Microsyst. 3, 011003 (2023). 10.1117/1.JOM.3.1.011003 38084130 PMC10712292

[27] Renkoski T. E. et al., “Ratio images and ultraviolet C excitation in autofluorescence imaging of neoplasms of the human colon,” J. Biomed. Opt. 18(1), 016005 (2013). JBOPFO 1083-3668 10.1117/1.JBO.18.1.016005 23291657 PMC3537599

[28] Banerjee B. et al., “Tryptophan autofluorescence imaging of neoplasms of the human colon,” J. Biomed. Opt. 17(1), 016003 (2012). 10.1117/1.JBO.17.1.016003 22352653

[29] Yamamoto N. Iga K., “Evaluation of gradient-index rod lenses by imaging,” Appl. Opt 19(7), 1101–1104 (1980). APOPAI 0003-6935 10.1364/AO.19.001101 20220993

[30] Drezek R. et al., “Autofluorescence microscopy of fresh cervical-tissue sections reveals alterations in tissue biochemistry with dysplasia,” Photochem. Photobiol. 73(6), 636–641 (2001). PHCBAP 0031-8655 10.1562/0031-8655(2001)0730636AMOFCT2.0.CO2 11421069

[31] Wu Y. Qu J. Y., “Autofluorescence spectroscopy of epithelial tissues,” J. Biomed. Opt. 11(5), 054023 (2006). JBOPFO 1083-3668 10.1117/1.2362741 17092172

[32] Solomon M. et al., “Optical imaging in cancer research: basic principles, tumor detection, and therapeutic monitoring,” Med. Princ. Pract. 20(5), 397–415 (2011). 10.1159/000327655 21757928 PMC7388590

[33] Wallace M. B. et al., “Reflectance spectroscopy,” Gastrointest. Endosc. Clin. N. Am. 19(2), 233–242 (2009). 10.1016/j.giec.2009.02.008 19423021 PMC2841958

